# Impact and Cost-Effectiveness of Biomedical Interventions on Adult Hepatitis B Elimination in China: A Mathematical Modelling Study

**DOI:** 10.1007/s44197-023-00132-1

**Published:** 2023-06-22

**Authors:** Xinran Wang, Zhicheng Du, Yijing Wang, Junren Wang, Shanshan Huang, Ying Wang, Jing Gu, Wanyu Deng, Stuart Gilmour, Jinghua Li, Yuantao Hao

**Affiliations:** 1 Department of Medical Statistics, School of Public Health, Sun Yat-sen University, Guangzhou, 510080 China; 2 Sun Yat-sen Global Health Institute, Sun Yat-sen University, Guangzhou, 510080 China; 3 Key Laboratory of Health Informatics of Guangdong Province, Sun Yat-sen University, Guangzhou, 510080 China; 4 Guangzhou Joint Research Center for Disease Surveillance, Early Warning and Risk Assessment, Guangzhou, 510080 China; 5 College of Life Science, Shangrao Normal University, Shangrao, 334001 China; 6 Graduate School of Public Health, St. Luke’s International University, Tokyo, Japan; 7 Peking University Center for Public Health and Epidemic Preparedness and Response, Beijing, 100191 China; 8 Key Laboratory of Epidemiology of Major Diseases (Peking University), Ministry of Education, Beijing, 100191 China

**Keywords:** Hepatitis B, Mathematical modeling, Cost-effectiveness, Elimination, Intervention

## Abstract

**Background:**

China has one of the highest hepatitis B virus (HBV) disease burdens worldwide and tracking progress toward the 2030 HBV elimination targets is essential. This study aimed to assess the impact of biomedical interventions (i.e., adult vaccination, screening and treatment) on the adult HBV epidemic, estimate the time for HBV elimination, and evaluate the cost-effectiveness of the interventions in China.

**Methods:**

A deterministic compartmental model was developed to project the HBV epidemic from 2022 to 2050 and estimate the time to meet elimination targets under four intervention scenarios. Cost-effectiveness was calculated using incremental cost per quality-adjusted life year (QALY) gained, i.e., average cost-effectiveness ratio (CER).

**Results:**

Under the status quo, there will be 42.09–45.42 million adults living with HBV in 2050 and 11.04–14.36 million HBV-related deaths cumulatively from 2022 to 2050. Universal vaccination would cumulatively avert 3.44–3.95 million new cases at a cost of US$1027–1261/QALY gained. The comprehensive strategy would cumulatively avert 4.67–5.24 million new chronic cases and 1.39–1.85 million deaths, expediting the realization of the elimination targets forward to 2049. This strategy was also cost-effective with an average CER of US$20,796–26,685/QALY and a saved healthcare cost of US$16.10–26.84 per person.

**Conclusion:**

China is not on track to meet the elimination targets but comprehensive biomedical interventions can accelerate the realization of the targets. A comprehensive strategy is cost-effective and cost-saving, which should be promoted in primary care infrastructures. Universal adult vaccination may be appropriate in the near future considering practical feasibility.

**Supplementary Information:**

The online version contains supplementary material available at 10.1007/s44197-023-00132-1.

## Introduction

An estimated 296 million people worldwide are chronically infected with hepatitis B virus (HBV) and 820,000 people die annually due to HBV-related diseases, disproportionately in low and middle-income countries (LMICs) [1]. The World Health Organization (WHO) published the Global Health Sector Strategy (GHSS) to eliminate viral hepatitis as a public health threat by 2030, with targets of reducing new chronic hepatitis B (CHB) infections by 95% and mortality by 65% compared with the 2015 baseline [2, 3]. China is the country experiencing the highest HBV disease burden, accounting for one-third of global HBV infections [4]. Therefore, China’s efforts to control HBV will have a significant impact on global elimination and provide inspiration for LMICs with a high burden of HBV infection [5].

China has reached the 2030 intervention targets for three-dose infant vaccination (90%) and timely birth-dose vaccination (90%) due to the early introduction of infant vaccination in 1992 and subsequent integration into the National Expanded Programme on Immunization in 2002 [6]. However, the highly effective vaccination is underutilized among adults—national and regional serology tests have found hepatitis B surface antigen (HBsAg) prevalence among adults was higher and hepatitis B antibody (HBsAb) prevalence was much lower than the level among juveniles [7, 8]. More than 99% of newly reported CHB cases have been populations aged over 15 years since 2015 [9, 10]. The United States Advisory Committee on Immunization Practices [11] and Hall et al. [12] recommended universal vaccination for adults regardless of their infection risk. Whereas some economic analyses only confirmed the cost-effectiveness of vaccination for high-risk populations, such as injection drug users [13] and men who have sex with men [14]. As adults bear the most of CHB disease burden and infant vaccination cannot control the spread in the general population, adult vaccination should be paid more attention in China.

Progress toward reaching the 2030 targets for diagnosis (19% versus the 2030 target of 90%), and treatment (11% versus the 2030 target of 80%) has also been slow [15]. China released the guidelines for primary care of CHB (2020), which proposed the CHB cascade of care and management, including screening and diagnosis, treatment, and follow-up [16]. However, failure to screening results in many infected individuals untreated. The attributable mortality will keep increasing without a significant scale-up in CHB treatment [3]. The lack of HBV awareness, limited healthcare budgets and weak political will were the main causes of the low intervention coverage [17]. To fill the intervention gap, the health and economic impact of these interventions are important information for effective resource allocation and objective decision-making. Although several studies have examined the cost-effectiveness of biomedical interventions for adults using the Markov model [18–20], they neglected disease transmission in the modeled cohort, which might underestimate the cost-effectiveness. Therefore, developing a new assessment tool is imperative.

Dynamic compartmental modelling is a valuable tool for analyzing disease transmission and health impacts of intervention strategies, so as to track progress towards the elimination targets at a population level [21]. Several modelling studies have investigated HBV in China, mostly highlighting the importance of infant and birth-dose vaccination on HBV epidemic [22–24]. But as far as we knew, no previous dynamic compartmental modelling studies assessed the impact of adult vaccination and its combinations with other interventions on HBV epidemic in China [19]. The aims of this study were to develop a compartmental model projecting CHB in Chinese adults, incorporating biomedical interventions including adult vaccination, screening, and treatment; to assess the impact of these interventions and their combinations on the future course of the HBV epidemic and elimination progress; and to illustrate the cost-effectiveness of intervention strategies.

## Methods

### Model Overview

A population-level, deterministic, dynamic age-structured transmission model was applied to explore the impact and cost-effectiveness of biomedical intervention strategies for Chinese population aged 18–80 years old from 2006 to 2050. We adopted most assumptions of previous dynamic models [25, 26] and fitted the model with the latest locally available Chinese epidemiological and demographic data.

The compartmental structure of the model is shown in Fig. S1. The general population are divided into 24 HBV-related health states, including susceptible (compartment S), acute HBV infection (compartment A), immunized (compartment I) and various phases of CHB infection. Populations in the susceptible compartment can transition into the immunized state upon vaccination. Initially infected members of the susceptible population enter the acute stage, and from there they can recover or progress into the first phase of CHB infection, labeled ${U}^{1}$ in Fig. S1. Modeled CHB phases are immune tolerant, immune active, immune control, immune reactivation, compensated cirrhosis (CC), decompensated cirrhosis (DC), and hepatocellular carcinoma (HCC) [27]. Immune tolerant and immune active are the most infective phases. The former six CHB phases can develop into HCC at a rate related to CHB phase and cascade of care. HBV-related death can occur in any of the CC, DC and HCC phases, at phase-specific rates. The diagram in Fig. S1 is ordered with columns to describe stages of disease, and rows representing the CHB cascade of care, which are undiagnosed, diagnosed but not on treatment, and on treatment. Within each row the seven stages of disease progression are labeled ${U}^{j}$ for undiagnosed, ${D}^{j}$ for diagnosed not in treatment, and ${T}^{j}$ for diagnosed and in treatment, for *j* = *1,…,6* and *HCC*. Disease progression and mortality rates are reduced following treatment [28]. The modeled population is dynamic, increasing because of births, and decreasing because of deaths (HBV-related and all-cause).

A system of partial differential equations (PDE) was used to formalize the flow between compartments, solved numerically in MATLAB 2020 (MathWorks, USA), using a difference equation method with a time step of one year. The model was projected from 2020 to 2050, and a range of epidemiological outcomes will be extracted under different policy scenarios. The detailed description of the model and PDEs is presented in the Supplementary Section 1.

### Data Source

We collected data from published literature, open access data and field investigation. The data included disease progression parameters, demographic data, HBV epidemiological data used for compartmental model, and costs and utilities used for cost-effectiveness analysis. Newly CHB cases reported by age from 2006 to 2018 were extracted from the National Notifiable Disease Reporting System (NNDRS) [10]. Birth rate and age-specific background mortality rates were obtained from the Chinese Statistical Yearbook and applied to simulate demographic changes at the country level. Detailed data is presented in Supplementary Section 1.4 and Sect. 2.

### Intervention Scenarios

To identify suitable strategy to achieve HBV elimination, we first modelled the epidemic under a *status quo* scenario, with intervention coverage remaining at the 2020 level. We then modelled four scenarios (Table 1) considering different combinations of screening, vaccination, and treatment. The *Universal vaccination* scenario simulated the implementation of HBV vaccination among all adults, increasing linearly from 20% in 2022 to 90% in 2030. In *Screening and vaccination* scenario, screening coverage increased to 90% and those tested HBsAb-negative are vaccinated. Scenario *Screening and treatment* modelled the situation where 90% patients were screened and 80% eligible for treatment received Tenofovir as recommended [29]. The *Comprehensive interventions* scenario included the combinations of 90% screening coverage, 90% adult immunization coverage and 80% treatment coverage for the eligible patients. We assumed lifetime protection of HBV vaccines and that screening is only implemented once in the cohort population’s lifetime. The coverage and efficacy of vaccine and antiviral drugs were set as parameters in the compartmental model to match different strategy scenarios.

**Table 1:** Intervention scenarios to simulate in the model

Scenarios	Percentage of relevant population covered^a^		
	Adult Hep3 vaccination	Screening	Treatment^b^
Status quo	20	19	11
Universal vaccination	90	19	11
Screening and vaccination	90	90	11
Screening and treatment	20	90	80
Comprehensive	90	90	80

^a^Linear scale-up to the target rate from 2022 to 2030

^b^WHO target: 90% diagnosed; 80% of people eligible for treatment receive antiviral treatment

### Elimination Analysis

We calculated the timeline needed to achieve the elimination targets under each scenario as the elimination analysis. Annual CHB incidence should be reduced by 95% compared to the 2015 baseline. There were two elimination thresholds for mortality: the relative elimination target is that the number of HBV-related deaths should be reduced by 65% compared to the 2015 baseline (simulated from the model); the absolute elimination target is that the HBV-related mortality rate should be reduced to ≤ 4/100,000 per year [30].

### Model Calibration

The model was calibrated against reported CHB incidence from 2006 to 2018 [10]. We first generated a sample of 1000 sets of parameter combinations randomly within ± 25% of model parameters using a beta(2,2) distribution. The 400 simulation outputs with the best goodness of fit based on the deviance criterion were selected as the sensitivity interval [31]. Details of calibration are given in Supplementary Section 5.

### Cost-Effectiveness Analysis

We conducted a cost-effectiveness analysis for all scenario strategies from a health providers perspective. Future costs and quality-adjusted life years (QALYs) per person were calculated with an annual discount rate of 3%, as recommended by the WHO in the Vaccine Health Economics Evaluation Guide [32]. All costs were reported in 2020 US Dollars and calculated using the International Monetary Fund gross domestic product (GDP) deflator and implied purchasing power parity conversion rates. The average cost-effectiveness ratio (CER) of each scenario relative to the status quo was calculated. The WHO definition of cost-effectiveness was applied: (1) strategies that cost less than the GDP per capita per QALY gained are very cost-effective; (2) those that cost between one and three times the GDP per capita per QALY gained are cost-effective [33].

### Sensitivity Analysis

We performed one-way sensitivity analysis to assess the influence of parameters and model robustness, which are investigated using tornado plots. Gamma distributions were used for sensitivity analysis of costs, beta distributions for progression rates, epidemiological parameters, and utilities, and a uniform distribution for the discount rate.

## Results

### Epidemic Projections Under Status Quo

**Fig. 1:**
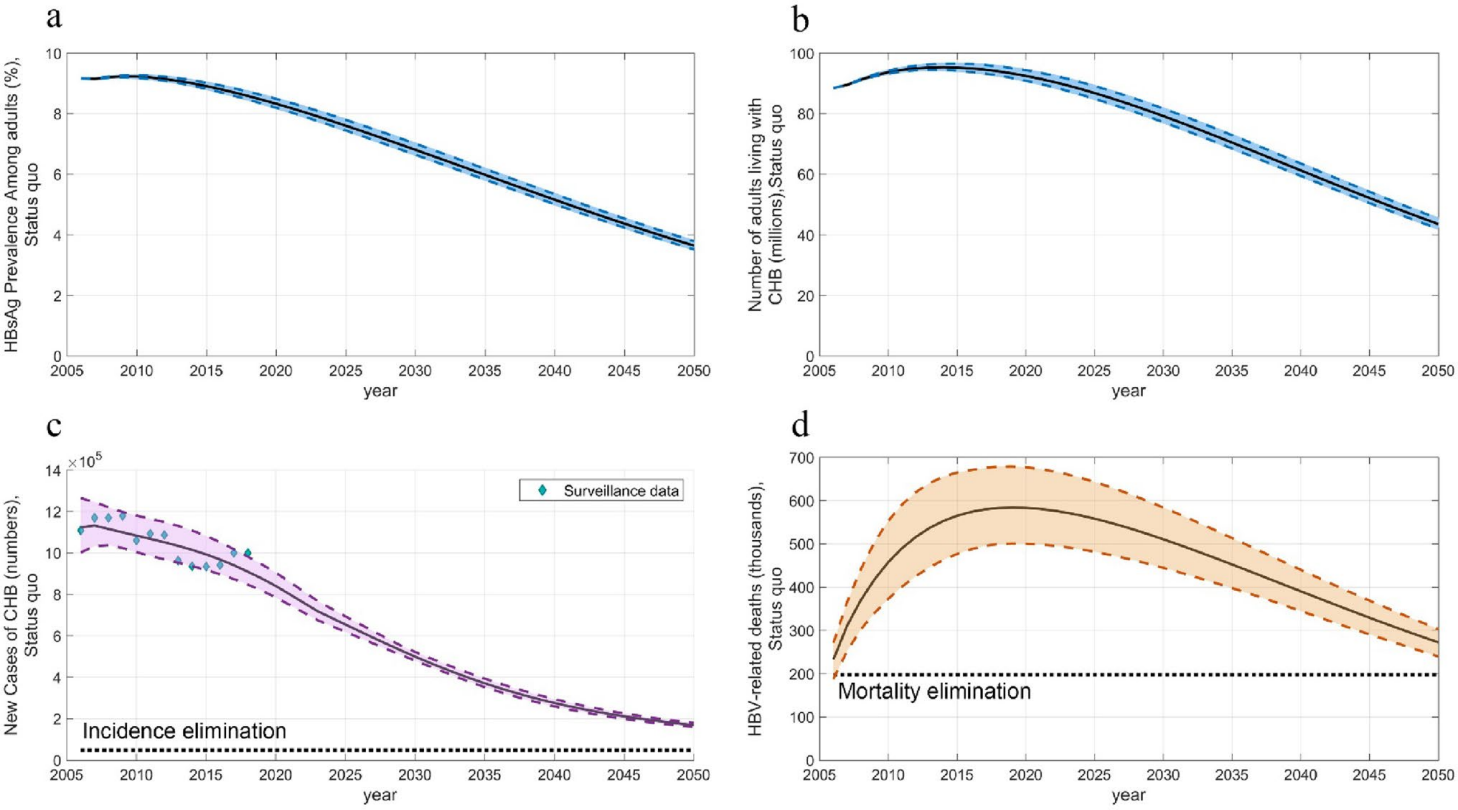
Epidemic under status quo. **a** HBsAg prevalence, **b** Number of people living with HBV, **c** CHB incidence, and **d** HBV-related deaths among adults. Shadow area with dotted lines border represent the uncertainty range. *HBV* hepatitis B virus, *CHB* chronic hepatitis B

The model estimates for the CHB incidence among Chinese adults showed good fit with national surveillance data. Under the status quo, the HBsAg prevalence among adults will continuously decrease to 6.81% (uncertainty range [UR] 6.65–7.01%) in 2030 and to 3.65% (UR 3.52–3.79%) in 2050 (Fig. 1a). There will be an estimated 79.25 (UR 77.24–81.73) million and 43.61 (UR 42.09–45.42) million people living with CHB respectively in 2030 and 2050 (Fig. 1b). New cases of CHB will gradually decline to 500,095 (UR 480,770–522,729) annually in 2030 and to 169,838 (UR 160,675–180,661) in 2050 (Fig. 1c). The threshold of incidence reduction of 95% the 2015 was set at 40,237, which was unlikely to be achieved by 2050.

The number of HBV-related deaths was 565,281 (UR 477,204–665,256) in 2015 and peaked at 584,288 (UR 477,204–679,232) in 2019. The relative elimination threshold of 35% the 2015 was set at 197,848. Although mortality began to decline after 2019, the relative mortality target is unlikely to be achieved by 2030, when mortality will reach 510,916 (UR 444,823–584,176) deaths annually (Fig. 1d), and this target will likely only be achieved after 2050. The absolute target (4/100,000) is also unachievable with a rate of 22.80/100,000 (UR 20.10–25.36/100,000) in 2050.

### Epidemiological Impact of Biomedical Interventions

**Fig. 2:**
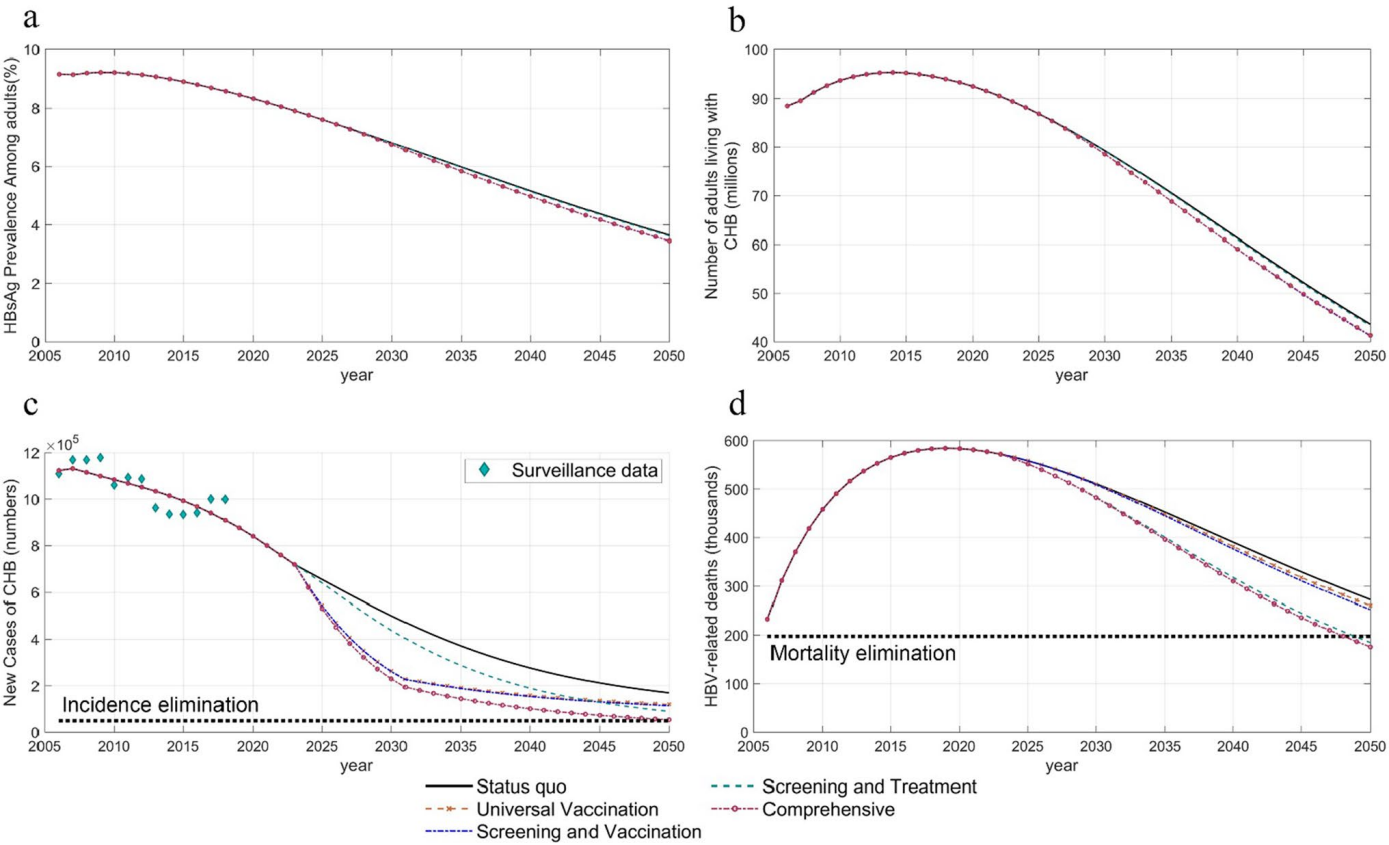
Impact of four community-based intervention scenarios on **a** HBsAg prevalence, **b** number of people living with HBV, **c** CHB incidence, and **d** HBV-related deaths among adults. *HBV* hepatitis B virus, *CHB* chronic hepatitis B

Figure 2 shows the impact of different intervention scenarios on CHB epidemic and mortality over time. Scenarios Universal vaccination and Screening and vaccination had similar impact on CHB epidemic and mortality. There will be 41.21 (UR 39.71–43.02) million and 41.22 (UR 39.73–43.02) million people living with CHB in the year 2050 under Universal vaccination and Screening and vaccination, respectively. The sole scale-up of vaccination would avert 3.69 (UR 3.44–3.95) million new CHB cases and 168,748 (UR 133,557–214,524) related deaths cumulatively over 28 years compared with the status quo. Screening and vaccination would avert 3.79 (UR 3.53–4.05) million new CHB cases and 271,230 (UR 221,457–326,595) million HBV-related deaths over 28 years. In 2050, the number of people living with CHB would be 43.29 (UR 41.89–45.03) million and 41.32 (UR 39.96–43.02) million under Screening and treatment and Comprehensive interventions scenarios, respectively. Screening and treatment would avert 1.90 (UR 1.79–2.00) million new CHB cases and 1.48 (UR 1.27–1.70) million HBV-related deaths over 28 years. Comprehensive interventions could cumulatively reduce 4.95 (UR 4.67–5.24) million new CHB patients and 1.61 (UR 1.39–1.85) million HBV-related deaths from 2022 to 2050 (Table 2).

**Table 2:** Epidemiological impact of combined interventions, 2022–2050

Scenarios	Prevalence in 2050 (%)	Cumulative new CHB cases	Cumulative HBV-related deaths	New CHB cases avoided	HBV-related deaths avoided
Status quo	3.65 (3.52–3.79)	11,390,623 (10,802,995–12,073,043)	12,631,977 (11,044,556–14,363,244)	–	–
Universal vaccination	3.45 (3.33–3.60)	7,697,266 (7,243,129–8,236,996)	12,463,229 (10,885,884–14,171,883)	3,693,357 (3,437,519–3,951,782)	168,748 (133,557–214,524)
Screening and vaccination	3.45 (3.33–3.60)	7,604,262 (7,152,447–8,139,258)	12,360,746 (10,792,881–14,053,070)	3,786,361 (3,528,353–4,047,218)	271,230 (221,457–326,595)
Screening and treatment	3.62 (3.50–3.76)	9,493,626 (8,999,353–10,090,860)	11,154,388 (9,720,003–12,675,729)	1,896,997 (1,789,343–1,999,092)	1,477,589 (1,273,280–1,697,738)
Comprehensive interventions	3.46 (3.35–3.59)	6,439,459 (6,027,553–6,911,208)	11,017,591 (9,589,462–12,527,800)	4,951,164 (4,667,557–5,243,163)	1,614,386 (1,391,040–1,852,562)

*CHB* chronic hepatitis B, *HBV* hepatitis B virus

**Fig. 3:**
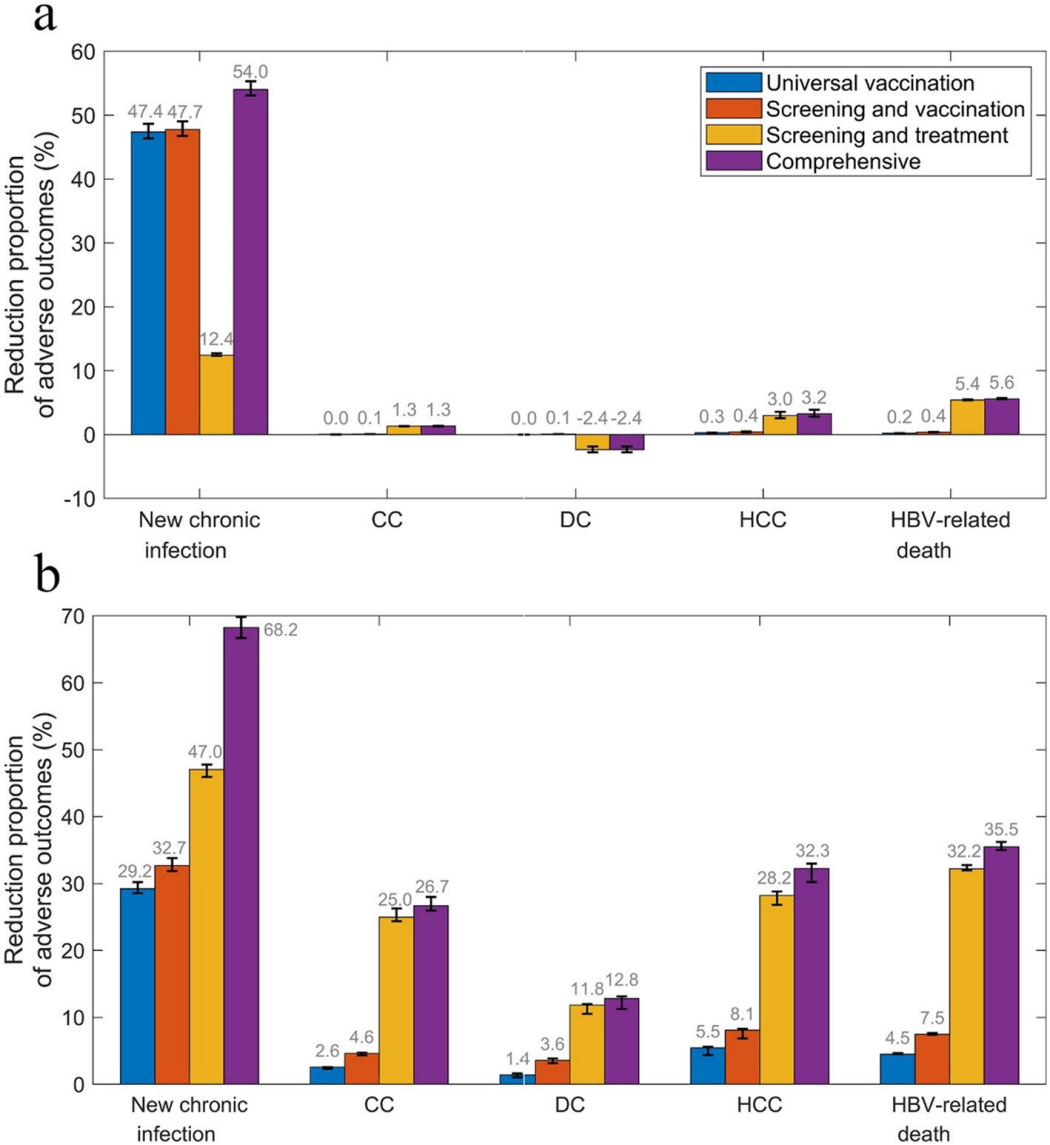
Reduction percentage of clinical outcomes of the scenarios compared to the status quo scenario in 2030 (**a**) and 2050 (**b**). The black error bar of each bar represents the uncertainty range of the reduction proportion. *HBV* hepatitis B virus, *CC* compensated cirrhosis, *DC* decompensated cirrhosis, *HCC* hepatocellular carcinoma

The impact of the four scenarios on clinical outcomes are shown in Fig. 3. Comparing Comprehensive interventions with the status quo, cases of new CHB, CC, DC, HCC and related deaths will be reduced by 54.0%, 1.3%, − 2.4%, 3.2% and 5.6% respectively in 2030; reduced by 68.2%, 26.7%, 12.8%, 32.3% and 35.5% respectively in 2050.

Year of achieving elimination targets for incidence was 2048 under Comprehensive strategy, while vaccination-led scenarios projected a much later timeline. Years of achieving elimination targets for mortality also varied between vaccination-led and treatment-led scenarios. The relative target of reducing 65% mortality will be achieved by 2056 under Universal vaccination (Table S5) and by 2055 under Screening and vaccination (Table S6). Meeting the absolute target of 4/100,000 will occur after 2080 for both scenarios. Under Screening and treatment, the time for the relative and absolute targets were 2049 and 2071, respectively (Table S7). Under Comprehensive interventions, the time for these two targets were 2049 and 2070, respectively (Table S8).

### Cost-Effectiveness Analysis

The costs and QALYs for all the intervention scenarios are shown in Table 3. Interventions conducted under the status quo would cost US$28.06 (UR 26.93–29.62) and yield 11.6335 (UR 11.6332–11.6337) QALYs per person from 2022 to 2050. Compared to the status quo, Universal vaccination generated 0.0059 (UR 0.0053–0.0065) incremental health benefits (i.e., QALYs) at an incremental cost of US$6.66 (UR 6.65–6.66). Moreover, a saved cost of US$8.78 (UR 8.13–9.36) per person in healthcare could offset 130–190% of the incremental cost. Screening and vaccination led to 0.0063 (UR 0.0056–0.0069) QALYs at an incremental cost of US$26.22 (UR 25.63–26.87). These two scenarios were very cost-effective, yielding an average CER for US$1123 (UR 1027–1261) and 4178 (UR 3805–4709)/QALY gained respectively, which were less than one times the GDP per capita (US$12,556).

**Table 3:** Benefits, costs and savings in healthcare costs of combined interventions, 2022–2050

Scenarios	QALY per person	Intervention cost ($) per person	Incremental QALY	Incremental intervention cost ($)	Average CER vs. status quo ($/QALY)	Health care cost ($) per person	Saved health care cost ($) per person
Status quo	11.6335 (11.6332–11.6337)	28.06 (26.93–29.62)	–	–	–	853.38 (792.56–915.92)	–
Universal vaccination	11.6394 (11.6394–11.6390)	34.72 (33.60–36.27)	0.0059 (0.0053–0.0065)	6.66 (6.65–6.66)	1123 (1027–1261)	844.60 (783.87–907.01)	8.78 (8.13–9.36)
Screening and vaccination	11.6398 (11.6397–11.6394)	54.28 (52.8–56.36)	0.0063 (0.0056–0.0069)	26.22 (25.63–26.87)	4178 (3805–4709)	844.24 (783.39–906.65)	9.14 (9.73–8.42)
Screening and treatment	11.6404 (11.6395–11.6409)	302.84 (295.56–312.31)	0.0070 (0.0056–0.0084)	274.78 (267.60–283.17)	39,427 (32,939–47,768)	838.70 (777.81–899.90)	14.68 (8.93–19.12)
Comprehensive interventions	11.6451 (11.6439–11.6456)	301.08 (293.84–310.37)	0.0116 (0.0102–0.0132)	273.02 (265.93–281.22)	23,536 (20,796–26,685)	831.31 (770.43–892.34)	22.07 (16.10–26.84)

*CHB* chronic hepatitis B, *QALY* quality-adjusted life years, *CER* cost-effectiveness ratio

Under the Screening and treatment scenario, 11.6404 (UR 11.6395–11.6409) QALYs can be gained at an intervention cost of US$302.84 (UR 295.56–312.31) and healthcare cost of US$838.70 (UR 777.81–899.90). Screening and treatment had an average CER larger than three times the GDP per capita, thus was not cost-effective but cost-saving. Under Comprehensive interventions, 11.6451 (UR 11.6439–11.6456) QALYs can be gained at an intervention cost of US$301.08 (UR 293.84–310.37) and healthcare cost of US$831.31 (UR 770.43–892.34). Comprehensive interventions were cost-effective with a CER of US$23,536 (UR 20,796–26,685), which were between one and three times the GDP per capita.

### Sensitivity Analysis

**Fig. 4:**
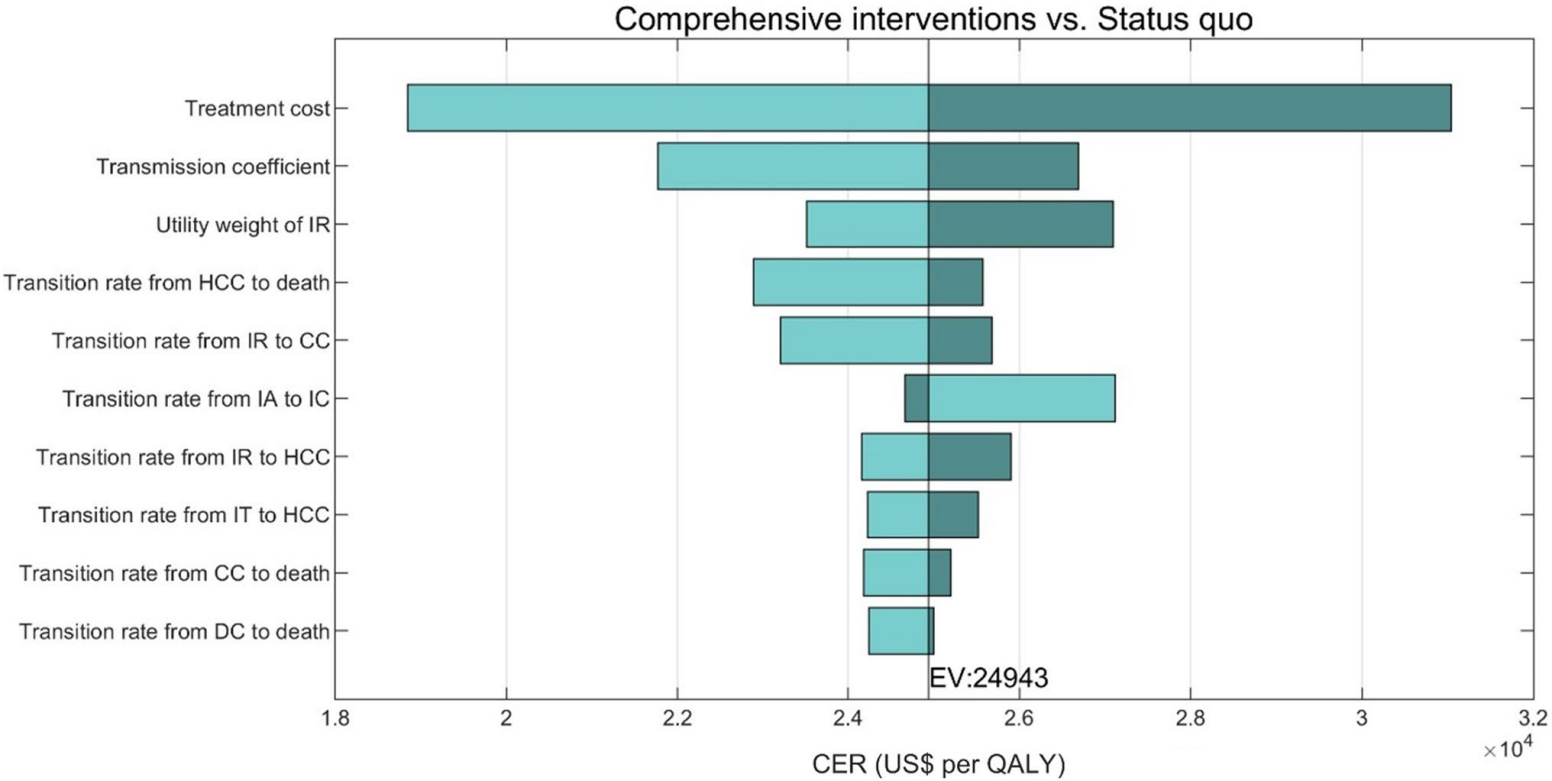
One-way sensitivity analysis of average CER (*Comprehensive interventions* vs. the Status quo). The dark green of each bar represents the high range of the parameter, and the light green part represents the low range. EV represents the CER for baseline parameters. *QALY* quality-adjusted life years, *IT* immune tolerant, *IA* immune active, *IC* immune control, *IR* immune reactivation, *CC* compensated Cirrhosis, *DC* decompensated Cirrhosis, *HCC* hepatocellular carcinoma

We analyzed the impact of the ten most influential parameters on average CER in tornado diagrams. The most influential factor of Comprehensive interventions is the cost of antiviral treatment (Fig. 4). If the cost of treatment was US$2060, which is 1.25 times higher than the base value, the CER would increase to its maximum value US$31,039/QALY gained; in contrast, if the cost was reduced to US$1236, 0.75 times the base value, the CER would be reduced to US$18,847/QALY gained. The one-way sensitivity analysis showed that the Comprehensive interventions scenario would remain cost-effective over the wide variety of all parameters. Vaccination cost was dominant in Universal vaccination; Screening and treatment costs were dominant in Screening and vaccination. Furthermore, treatment cost was also dominant in Screening and treatment (Fig. S5). Reducing the treatment cost to 75% of the base value would facilitate the transition of Screening and treatment from a non-cost-effective status to a cost-effective one.

## Discussion

This study developed a compartmental mathematical model to predict the future trends of the HBV epidemic among adults, and to evaluate the effect and cost-effectiveness of biomedical interventions in China. Our study demonstrated that scale-up comprehensive interventions is cost-saving for averting 4.95 million new CHB cases and 1.61 million HBV-related deaths. Adult vaccination is also cost-effective mainly for averting 3.69 million new CHB cases cumulatively from 2022 to 2050. The implementation of biomedical interventions will accelerate the time for realization of the elimination targets.

The modeling results showed the burden of CHB, including the number of people living with CHB, new cases of CHB and attributable deaths, will gradually decrease from 2022 to 2050. Our modeling results are essentially consistent with a previous study [34], which predicted that there will be 60 million people living with HBV in 2030 and 10 million HBV-related deaths from 2015 to 2030 without further interventions. The decreasing trend over time could be partly explained by natural aging of CHB people and China’s previous efforts on infant vaccination. In line with the findings of Su et al. [19] China cannot meet the WHO relative target to reduce 65% HBV-related deaths on time, i.e., by 2030. Even the most optimistic comprehensive interventions can only bring the time forward eight years to 2049 to meet the goal, compared with 2057 under current practice. In June 2021, WHO released an interim guidance simplifying the elimination targets into absolute ones, aiming for direct comparison of progress towards elimination and reliable validation process [30]. According to the new guideline, an annual mortality rate of 4/100,000 by 2030 is set as the new target. But this new target is ambitious and unrealistic for China with a high burden of CHB and large population base. This study is a fresh evaluation of the application of both the relative and the absolute targets, and verifies the potential limitation mentioned in the interim guideline for the new target. Countries with high disease burdens are more likely to achieve relative goals, while those with low burdens are expected to meet the absolute target [35]. To help measure progress toward true elimination, different thresholds of the absolute target might be set according to HBV epidemic conditions and service coverage initiatives of countries.

The analysis suggested that comprehensive interventions are cost-effective, resulting in better health outcomes by reducing the cases of new chronic infections, CC, DC, HCC, and attributable deaths. This is in line with previous cost-effectiveness analyses at population level in different settings. Xu et al. indicated comprehensive intervention was cost-effective at an ICER of US$420/QALY gained at 100% screening intensity in rural China with high prevalence [18]. Chahal et al. found the same strategy cost-effective among six high-risk/prevalence populations in America at ICER ranging from $3203–$18,009 per QALY gained [14]. Screening identifies people who are HBsAb negative or need medical management. Subsequent vaccination and treatment are efficient in preventing infections and slowing CHB disease progression to LC and HCC, therefore significantly averting morbidity and mortality. However, the real-world practicality of the strategy is important to consider for the future progress. Su et al. referred the infeasibility of mass screening due to deficient training of PCs and financial funding, as well as inadequate utilization of the primary care infrastructure in community health centers (CHCs) [19]. Our sensitivity analysis revealed that the cost of treatment was a critical factor on the cost-effectiveness of treatment-led scenarios. The findings of similar studies [36–38] also acknowledged the high price tag of antiviral treatment and its significant impact on the ICERs.

Fortunately, the comprehensive strategy is expected to quicken the pace of achieving the intervention coverage targets in China. Surveys showed 80% CHCs have the facilities required for follow-up and monitoring, and more than 90% of general practitioners are willing to participate in CHB management [39]. Moreover, patients were willing to receive community management and be referred to tertiary hospitals if needed. One of the HBV management pilots in Sichuan Health Service Center reported 100% patient satisfaction among 577 CHB patients under management [40]. As HBV cannot be cured and life-long antiviral therapy is required, reducing treatment expenses is crucial for enhancing the cost-effectiveness, ensuring the feasibility of treatment-led strategies, and providing valuable insights for healthcare policy and resource allocation. The prices for treatment have decreased after antiviral drugs were included in the National List of Reimbursable Medicines in 2017 [41]. Entecavir and tenofovir, the two first-line medicine recommended by WHO, were later selected in the Centralized Joint Procurement for generic drugs in 2018 [42] and 2022 [43], resulting in more affordable prices and widespread use. Still, it will take a long time for the effective implementation of the biomedical interventions. In details of actions, sustainable funding, price management of treatment, medical personnel development, and promotion of HBV health education are urgently required for the community management programs.

Our model also indicated that universal vaccination among adults is very cost-effective at the population level regardless of prior screening. Scenario Universal vaccination, which aims to vaccinate the whole population, would essentially increase vaccine coverage among people who are HBsAb-negative, thereby averting new chronic infections. In contrast, Screening and vaccination aims to identify those susceptible by universal screening and then vaccinating accordingly. In addition to the inherent benefits of vaccinating the susceptible, this strategy has the potential to raise awareness of infection status among individuals infected with HBV. Therefore, these diagnosed HBV-positive individuals would enter treatment at the rate of base case, which would result in some marginal health benefit in reducing CHB epidemic and mortality. Improved engagement with treatment also made the strategy sensitive to treatment cost in sensitivity analysis. However, vaccination-led strategies were dominated in Xu et al. [18] and Hutton et al. [44], whose Markov models ignored real-world transmission dynamics or were conducted among populations with a low prevalence, underestimating the effect of vaccines. Regarding the continually high CHB incidence among adults, our findings supported the expansion of a universal vaccination program for better feasibility and cost-effectiveness. HBV screening was removed from routine health examinations in 2010 to avoid HBV discrimination [45]. Implementing such a universal vaccination program should improve residents’ accessibility and compliance to get vaccinated. Adults could also be organized by their educational institutions or working organizations to get vaccinated, which, to a certain extent, avoids HBV discrimination and other technical issues caused by screening [46]. The quick benefit of reducing new CHB cases would lay the foundation for the implementation of comprehensive interventions and elimination progress.

This study helps us understand the roles of biomedical intervention strategies in China’s HBV elimination progress among adults and provide perspectives on HBV resource allocation optimization for policymakers. However, this study has some limitations. First, we only applied the model to a national level and blurred the difference between provincial epidemic situations. But it can be viewed as an example of model application, which may be applied in future work to explore situations at subnational levels. Second, unavailability and uncertainty of epidemiological data impeded the precise analysis of specific population such as HBV-HIV/HCV co-infected patients or pregnant woman. This suggested that the monitoring and quality control system should be strengthened to obtain high-quality and all-round HBV data. Third, we did not include other recommended antivirals, nor the adverse effect of long-term prescription in the model, but this issue was alleviated by the sensitivity analysis to a certain extent. Finally, we considered only direct medical care and intervention costs from a healthcare provider perspective, and did not detail indirect cost components due to data availability. Further studies are warranted when detailed costs data available.

## Conclusion

In conclusion, we proposed a mathematical model to track 2030 HBV elimination targets in China and assess the cost-effectiveness of comprehensive biomedical interventions. Although China is not on track to complete the elimination targets, the comprehensive interventions including adult vaccination, screening and treatment can reduce loss of lives and bring the realization of elimination ahead, inspiring further action to promote the implementation in China. In addition, universal vaccination may also be appropriate in the near future considering the effectiveness for reducing incidence and the practical feasibility.

## Supplementary Information

Below is the link to the electronic supplementary material.

Supplementary file1 (PDF 1083 KB)

## Data Availability

All data used in this study are available in the article or the Supplementary. Codes were obtained at the request of the corresponding author.

## Abbreviations

HBV: Hepatitis B virus
WHO: World Health Organization
LC: Liver cirrhosis
HCC: Hepatocellular carcinoma
GHSS: Global Health Sector Strategy
HBsAg: Hepatitis B surface antigen
CHB: Chronic hepatitis B
ACIP: The United States Advisory Committee on Immunization Practices
GP: General practitioner
CHC: Community health center
IT: Immune tolerant
IA: Immune active
IC: Immune control
IR: Immune reactivation
CC: Compensated cirrhosis
DC: Decompensated cirrhosis
PDE: Partial differential equation
QALY: Quality-adjusted life years
GDP: Gross domestic product
CER: Cost-effectiveness ratio
UR: Uncertainty range
CER: Cost effectiveness ratio
