# Supplementary material for: Impact and Cost-Effectiveness of Biomedical Interventions on Adult Hepatitis B Elimination in China: A Mathematical Modelling Study
Source: J Epidemiol Glob Health. 2023 Jun 22;13(3):517–27. doi: 10.1007/s44197-023-00132-1 (PMC10469118; doi:10.1007/s44197-023-00132-1)
Supplement: Supplementary file 1 — Supplementary file1 (PDF 1083 KB) [file 44197_2023_132_MOESM1_ESM.pdf]

Supplementary material:

Impact and cost-effectiveness of biomedical interventions on adult hepatitis B elimination in China: A mathematical modelling study

Xinran Wang,[1,2,3,¶] Zhicheng Du,[1,2,3,¶] Yijing Wang,[1,2,3] Junren Wang,[1] Shanshan Huang,[1] Ying Wang,[1,2,3] Jing Gu,[1,2,3,4] Wanyu Deng,[5] Stuart Gilmour,[6] Jinghua Li,[1,2,3,4*] Yuantao Hao[7,8,*]

[1]Department of Medical Statistics, School of Public Health, Sun Yat-sen University, Guangzhou 510080, China.

[2]Sun Yat-sen Global Health Institute, Sun Yat-sen University, Guangzhou 510080, China.

[3]Key Laboratory of Health Informatics of Guangdong Province, Sun Yat-sen University, Guangzhou 510080, China.

[4]Guangzhou Joint Research Center for Disease Surveillance, Early Warning and Risk Assessment, Guangzhou 510080, China.

[5]College of Life Science, Shangrao Normal University, Shangrao 334001, China.

[6]Graduate School of Public Health, St. Luke's International University, Tokyo, Japan.

[7]Peking University Center for Public Health and Epidemic Preparedness & Response, Beijing 100191, China.

[8]Key Laboratory of Epidemiology of Major Diseases (Peking University), Ministry of Education, Beijing 100191, China.

[¶]Xinran Wang and Zhicheng Du have contributed equally.

***Correspondence to**

Jinghua Li, *lijinghua3@mail.sysu.edu.cn*

Department of Medical Statistics, School of Public Health, Sun Yat-sen Unveristy, Guangzhou 510080, China

Yuantao Hao, *haoyt@bjmu.edu.cn*, ORCID 0000-0003-4146-9262

Peking University Center for Public Health and Epidemic Preparedness & Response, Beijing 100191, China

1 Model overview 4
1.1 Model framework 4
1.2 Partial differential equations 4
1.3 Force of infection 6
1.4 Key model parameters 6
2 Epidemiological data and initial values 10
2.1 Epidemiological data 10
2.2 Initial values 11
3 Intervention scenarios 12
4 Model outputs 13
4.1 Epidemiological outcomes 13
4.2 Economic outcomes 14
5 Model calibration 15
6 Supplemental results 16
6.1 Epidemic projections 16
6.2 Cost-effectiveness analysis 18
7 CHEERS checklist 18
8 References 20

Supplementary tables

Table S1 Health status in the model (Page4)

Table S2 Parameters in the compartmental model (Page7)

Table S3 HBV Epidemiological data (Page10)

Table S4 Initial population in compartments (Page12)

Table S5 Elimination analyses under Universal vaccination (Page16)

Table S6 Elimination analysis under Screening and vaccination (Page17)

Table S7 Elimination analysis under Screening and treatment (Page17)

Table S8 Elimination analyses under Comprehensive interventions (Page18)

Table S9 CHEERS checklist (Page18)

Supplementary figures (at the end of the file)

Fig S1. HBV compartmental model diagram with parameters

Fig S2. Impact (with uncertainty range) of four community-based intervention scenarios on (a) HBsAg prevalence, (b) Number of people living with HBV, (c) CHB incidence, and (d) HBV-related deaths among adults.

Fig S3. One-way sensitivity analysis of average CER (Universal vaccination vs. the Status quo).

Fig S4. One-way sensitivity analysis of average CER (Screening and vaccination vs. the Status quo)

Fig S5. One-way sensitivity analysis of average CER (Screening and treatment vs. the Status quo).

1 Model overview

1.1 Model framework

Fig. S1 shows the compartmental structure of the model, with each box showing a population group and the arrows describing flows between population groups. Greek symbols above or next to the arrows show transition rates. Movement between the states is specified by partial differential equations. The state variables are represented by $X(a,t)$, which X represents infection state (Table S1) in terms of age (a) and time (t). Transition rates and model parameters are given in Table S2.

The modelled population is divided into 24 different health states to describe the natural history of chronic hepatitis B over time. Table S1 describes these health states. CHB cascade of care is denoted as U^j (infected but undiagnosed), D^j (diagnosed), T^j (diagnosed and on treatment), for j=1,…,6 and HCC. The superscripts j of U, D, and T compartments represent CHB phases.

Table S1 Health status in the model

Status labels	Status NO.	Definition
S	1	Susceptible
A	2	Acute HBV infection
I	3	Immunized
U^1, D^1, T^1	4,5,6	Immune Tolerant of cascade of care
U^2, D^2, T^2	7,8,9	Immune Active of cascade of care
U^3, D^3, T^3	10,11,12	Immune Control of cascade of care
U^4, D^4, T^4	13,14,15	Immune Reactivation of cascade of care
U^5, D^5, T^5	16,17,18	Compensated Cirrhosis of cascade of care
U^6, D^6, T^6	19,20,21	Decompensated Cirrhosis of cascade of care
U^{HCC}, D^{HCC}, T^{HCC}	22,23,24	Hepatocellular Carcinoma of cascade of care
M	25	HBV-related death

1.2 Partial differential equations

$$\frac{\partial S}{\partial a}+\frac{\partial S}{\partial t}=-S\cdot(\lambda(a,t)+p(a,t)+\mu^{nat}(a,t)) \quad (1)$$

$$\frac{\partial A}{\partial a}+\frac{\partial A}{\partial t}=S\lambda(a,t)-A\cdot(\sigma(a)+\theta(a)+\mu_0+\mu^{nat}(a,t)) \quad (2)$$

$$\frac{\partial I}{\partial a}+\frac{\partial I}{\partial t}=Sp(a,t)+A\theta(a,t)-I\cdot\mu^{nat}(a,t) \quad (3)$$

$$\frac{\partial U^1}{\partial a}+\frac{\partial U^1}{\partial t}=A\sigma(a)-U^1\cdot(r_1^U+r_2^U+\delta^1+\mu^{nat}(a,t)) \tag{4}$$

$$\frac{\partial D^1}{\partial a}+\frac{\partial D^1}{\partial t}=U^1\delta^1-D^1\cdot(r_1^D+r_2^D+\varphi^1+\mu^{nat}(a,t)) \tag{5}$$

$$\frac{\partial T^1}{\partial a}+\frac{\partial T^1}{\partial t}=D^1\varphi^1-T^1\cdot(r_1^T+r_2^T+\mu^{nat}(a,t)) \tag{6}$$

$$\frac{\partial U^2}{\partial a}+\frac{\partial U^2}{\partial t}=U^1r_1^U-U^2\cdot(r_3^U+r_4^U+r_5^U+r_6^U+\delta^2+\mu^{nat}(a,t)) \tag{7}$$

$$\begin{aligned}\frac{\partial D^2}{\partial a}+\frac{\partial D^2}{\partial t}=&\,D^1r_1^D+U^2\delta^2-D^2\\&\cdot(r_3^D+r_4^D+r_5^D+r_6^D+\varphi^2+\mu^{nat}(a,t))\end{aligned} \tag{8}$$

$$\frac{\partial T^2}{\partial a}+\frac{\partial T^2}{\partial t}=D^2\varphi^2+T^1r_1^T-T^2\cdot(r_3^T+r_4^T+r_5^T+r_6^T+\mu^{nat}(a,t)) \tag{9}$$

$$\frac{\partial U^3}{\partial a}+\frac{\partial U^3}{\partial t}=U^2r_3^U-U^3\cdot(r_7^U+r_8^U+\delta^3+\mu^{nat}(a,t)) \tag{10}$$

$$\frac{\partial D^3}{\partial a}+\frac{\partial D^3}{\partial t}=D^2r_3^D+U^3\delta^3-D^3\cdot(r_7^D+r_8^D+\varphi^3+\mu^{nat}(a,t)) \tag{11}$$

$$\frac{\partial T^3}{\partial a}+\frac{\partial T^3}{\partial t}=D^3\varphi^3+T^2r_3^T-T^3\cdot(r_7^T+r_8^T+\mu^{nat}(a,t)) \tag{12}$$

$$\frac{\partial U^4}{\partial a}+\frac{\partial U^4}{\partial t}=U^2r_4^U+U^3r_7^U-U^4\cdot(r_9^U+r_{10}^U+\delta^4+\mu^{nat}(a,t)) \tag{13}$$

$$\begin{aligned}\frac{\partial D^4}{\partial a}+\frac{\partial D^4}{\partial t}=&\,D^2r_4^D+D^3r_7^D+U^4\delta^4-D^4\\&\cdot(r_9^D+r_{10}^D+\varphi^4+\mu^{nat}(a,t))\end{aligned} \tag{14}$$

$$\frac{\partial T^4}{\partial a}+\frac{\partial T^4}{\partial t}=D^4\varphi^4+T^2r_4^T+T^3r_7^T-T^4\cdot(r_9^T+r_{10}^T+\mu^{nat}(a,t)) \tag{15}$$

$$\frac{\partial U^5}{\partial a}+\frac{\partial U^5}{\partial t}=U^2r_5^U+U^4r_9^U-U^5\cdot(r_{11}^U+r_{12}^U+\delta^5+\mu_1^u+\mu^{nat}(a,t)) \tag{16}$$

$$\begin{aligned}\frac{\partial D^5}{\partial a}+\frac{\partial D^5}{\partial t}=&\,D^2r_5^D+D^4r_9^D+U^5\delta^5-D^5\\&\cdot(r_{11}^D+r_{12}^D+\varphi^5+\mu_1^D+\mu^{nat}(a,t))\end{aligned} \tag{17}$$

$$\frac{\partial T^5}{\partial a}+\frac{\partial T^5}{\partial t}=D^5\varphi^5+T^2r_5^T+T^4r_9^T-T^5\cdot(r_{11}^T+r_{12}^T+\mu_1^T+\mu^{nat}(a,t)) \tag{18}$$

$$\frac{\partial U^6}{\partial a}+\frac{\partial U^6}{\partial t}=U^5r_{11}^U-U^6\cdot(r_{13}^U+\delta^6+\mu_2^U+\mu^{nat}(a,t)) \tag{19}$$

$$\frac{\partial D^6}{\partial a}+\frac{\partial D^6}{\partial t}=D^5r_{11}^D+U^6\delta^6-D^6\cdot(r_{13}^D+\varphi^6+\mu_2^D+\mu^{nat}(a,t)) \tag{20}$$

$$\frac{\partial T^6}{\partial a}+\frac{\partial T^6}{\partial t}=D^6\varphi^6+T^5r_{11}^T-T^6\cdot(r_{13}^T+r_{14}+\mu_2^T+\mu^{nat}(a,t)) \tag{21}$$

$$\frac{\partial U^{HCC}}{\partial a}+\frac{\partial U^{HCC}}{\partial t} = U^1 r_2^U + U^2 r_6^U + U^3 r_8^U + U^4 r_{10}^U + U^5 r_{12}^U + U^6 r_{13}^U - U^{HCC}\cdot(\delta^7 + \mu_3^U + \mu^{nat}(a,t)) \quad (22)$$

$$\frac{\partial D^{HCC}}{\partial a}+\frac{\partial D^{HCC}}{\partial t} = D^1 r_2^D + D^2 r_6^D + D^3 r_8^D + D^4 r_{10}^D + D^5 r_{12}^D + D^6 r_{13}^D + T^1 r_2^T + T^2 r_6^T + T^3 r_8^T + T^4 r_{10}^T + T^5 r_{12}^T + T^6 r_{13}^T + U^{HCC}\delta^7 - D^{HCC}\cdot(r_{15} + \mu_3^D + \mu^{nat}(a,t)) \quad (23)$$

$$\frac{\partial LT}{\partial a}+\frac{\partial LT}{\partial t} = D^{HCC} r_{15} + T^6 r_{14} - LT\cdot(\mu_3^T + \mu^{nat}(a,t)) \quad (24)$$

Therefore, the rate of change of HBV-related deaths *M* is as followed:

$$\frac{\partial M}{\partial a}+\frac{\partial M}{\partial t} = A\mu_0 + \sum_{i=1}^{3}\sum_{j=1}^{3}\left\{\begin{pmatrix} U^5 & U^6 & U^{HCC} \\ D^5 & D^6 & D^{HCC} \\ T^5 & T^6 & T^{HCC} \end{pmatrix}\cdot\begin{pmatrix} \mu_1^U & \mu_2^U & \mu_3^U \\ \mu_1^D & \mu_2^D & \mu_3^D \\ \mu_1^T & \mu_2^T & \mu_3^T \end{pmatrix}\right\} \quad (25)$$

where *i* and *j* represent rows and columns of the Hadamard product in brace.

1.3 Force of infection

Horizontal transmission is denoted by $\lambda(a,t)$ where

$$\lambda(a,t) = \beta_{all}\cdot\frac{\int_{18}^{80}\sum_{i\in\eta}\varepsilon_i X(a,t)da}{\int_{18}^{80}\sum_{i\in\forall}X(a,t)da} \quad (26)$$

where η consists of the indices of the infectious compartments

A, U^1, U^2, U^3, U^4, U^5, U^6, U^{HCC}, D^1, D^2, D^3, D^4, D^5, D^6 and D^{HCC}

where $\forall$ consists of the indices of the population compartments (all compartments) S, I, A, U^1, U^2, U^3, U^4, U^5, U^6, U^{HCC}, D^1, D^2, D^3, D^4, D^5, D^6, D^{HCC}, T^1, T^2, T^3, T^4, T^5, T^6 and T^{HCC}

1.4 Key model parameters

Table S2 Parameters in the compartmental model

Parameters	Definition	Value	References
**Annual transition rates**			

λ	From susceptible to acute (force of infection)	Shown in Section 1.3	-
p	From susceptible to immunized	Varies with vaccine efficacy and coverage	-
σ	From acute to immune tolerant (proportion of developing chronic infection)	$\exp(-0.645a^{0.455})$<br>The rate derived for each age was averaged to obtain age-group progression rates.	[1, 2]
θ	From acute to immunized	$2[1-\sigma(a)]$	[3]
r_1^k	From immune tolerant to immune active	0.0464 (for infected)<br>0.0464 (for diagnosed)<br>0.002 (for treated)	[4]
r_2^k	From immune tolerant to HCC	0.0073 (for infected)<br>0.0073 (for diagnosed)<br>0.0033 (for treated)	[4]
r_3^k	From immune active to immune control	0.078 (for infected)<br>0.078 (for diagnosed)<br>0.158 (for treated)	[5]
r_4^k	From immune active to immune reactivation	0.005 (for infected)<br>0.005 (for diagnosed)<br>0.0025 (for treated)	[6]
r_5^k	From immune active to CC	0.016 (for infected)<br>0.016 (for diagnosed)<br>0 (for treated)	[7]
r_6^k	From immune active to HCC	0.004 (for infected)<br>0.004 (for diagnosed)<br>0.003 (for treated)	[8]
r_7^k	From immune control to immune reactivation	0.01 (for infected)<br>0.01 (for diagnosed)<br>0 (for treated)	[9]
r_8^k	From immune control to HCC	0.00064 (for infected)<br>0.00064 (for diagnosed)<br>0.00048 (for treated)	[10]
r_9^k	From immune reactivation to CC	0.0078 (for infected)<br>0.0078 (for diagnosed)<br>0 (for treated)	[2]
r_{10}^k	From immune reactivation to HCC	0.0072 (for infected)<br>0.0072 (for diagnosed)<br>0.0035 (for treated)	[7]
r_{11}^k	From CC to DC	0.033 (for infected)<br>0.033 (for diagnosed)<br>0.0165 (for treated)	[2]
r_{12}^k	From CC to HCC	0.018 (for infected)<br>0.018 (for diagnosed)	[2]

		0.009 (for treated)	
r_{13}^k	From DC to HCC	0.048 (for infected)<br>0.048 (for diagnosed)<br>0.024 (for treated)	[2]
μ_1^k	From CC to HBV-related death	0.024 (for infected)<br>0.024 (for diagnosed)<br>0.012 (for treated)	[4]
μ_2^k	From DC to HBV-related death	0.075 (for infected)<br>0.075 (for diagnosed)<br>0.0375 (for treated)	[4]
μ_3^k	From HCC to HBV-related death	0.33 (for infected)<br>0.33 (for diagnosed)<br>0.2475 (for treated)	[2]
μ_0	From acute infection to death	0.001	[3]
δ^n	From infected U^n to diagnosed D^n, coverage of diagnoses	Varies with diagnostic coverage.	-
φ^n	From diagnosed D^n to treatment T^n, coverage of treatment	Varies with treatment coverage.	-
**Demographic characteristics**			
Initial population aged 18-80		964,898,000	[11]
μ^{nat}	Annual background mortality rate in 2006	0.00681	[11]
ρ	Annual maturation rate*	0.021	Calculated[11]
f	Annual entry rate*	0.0259	Calculated[11]
**Transmission parameters**			
ε_0	Infectiousness multiplier for acute infections	1	
ε_1^k	Infectiousness multiplier for immune tolerant	10 (for infected)<br>10 (for diagnosed)<br>0 (for treated)	
ε_2^k	Infectiousness multiplier for immune active	10 (for infected)<br>10 (for diagnosed)<br>0 (for treated)	
ε_3^k	Infectiousness multiplier for immune control	1 (for infected)<br>1 (for diagnosed)<br>0 (for treated)	[6]
ε_4^k	Infectiousness multiplier for immune reactivation	1 (for infected)<br>1 (for diagnosed)<br>0 (for treated)	
ε_5^k	Infectiousness multiplier for CC	1 (for infected)<br>1 (for diagnosed)<br>0 (for treated)	

ε_6^k	Infectiousness multiplier for DC	1 (for infected)<br>1 (for diagnosed)<br>0 (for treated)	
ε_7^k	Infectiousness multiplier for HCC	1 (for infected)<br>1 (for diagnosed)<br>0 (for treated)	
β	Transmission coefficient among adults	0.03	
**Intervention efficacy**			
v	Efficacy of HBV vaccine against HBV	0.95	[12]
**Utilities (annual)**			
Susceptible/ Immunized		1	
Acute infection		0.99	
Immune Tolerant		0.92 (for infected)<br>0.92 (for diagnosed)<br>0.99 (for treated)	
Immune Active		0.863 (0.85-0.88) (for infected)<br>0.863 (0.85-0.88) (for diagnosed)<br>0.98 (for treated)	
Immune Control		0.97 (for infected)<br>0.97 (for diagnosed)<br>0.98 (for treated)	
Immune Reactivation		0.863 (0.85-0.88) (for infected)<br>0.863 (0.85-0.88) (for diagnosed)<br>0.98 (for treated)	[4, 13, 14]
Compensated Cirrhosis		0.85 (0.83-0.87) (for infected)<br>0.85 (0.83-0.87) (for diagnosed)<br>0.95 (for treated)	
Decompensated Cirrhosis		0.40 (0.36-0.45) (for infected)<br>0.40 (0.36-0.45) (for diagnosed)<br>0.80 (for treated)	
Hepatocellular Carcinoma		0.44 (0.42-0.47) (for infected)<br>0.44 (0.42-0.47) (for diagnosed)	
Liver transplant		0.697 (0.69–0.71)	
**Costs (annual)**			
r	Discount rate	0.03	-
**Medical costs in health states (**$USD, 2020**)**			
Immune Tolerant		449	[15]
Immune Active		1478	[13]
Immune Control		449	[15]
Immune Reactivation		1478	[13]

Compensated Cirrhosis	1978	[13]
Decompensated Cirrhosis	3986	[13]
Hepatocellular Carcinoma	5883	[13]
Liver transplant	39433	[15]
**Intervention costs (**$USD, 2020**)**		
Annual vaccination cost	18.77	Estimated**
Annual screening cost	4.64	Estimated**
Annual diagnoses cost	93.86	Estimated**
Annual treatment cost (TDF)	1648	Estimated**

*: The entry rate is defined as the rate at which people enter the target group and the maturation rate is the sum of the background mortality rate and the rate of aging, both derived from the demographic data for China[16].

Entry rates: $\rho = -ln(1 - \frac{\text{18 years old population}}{\text{18-80 years old population}}) + \text{birth rate}$

Maturation rates: $b = -ln(1 - \frac{\text{80years old population}}{\text{18-80 years old population}}) + \text{mortality rate}$

**: Estimated based on field investigation.

2 Epidemiological data and initial values

2.1 Epidemiological data

China-specific data were incorporated into the model including demographic data, HBV serological data, historical intervention coverage, and cost data (already shown in Table S2). Data descriptions were as follows:

1. Demographic data comprised the annual population, annual birth rate, and background mortality rate. These data were collected from World Population Prospects 2022.
2. HBV epidemiological data for initial values and model calibration were listed in Table S3.

Table S3 HBV Epidemiological data

Variable	Value	Source
**Data for initial values**		
**HBsAg** (%)		
20~	12.00 (6.30-17.71)	
25~	8.99 (6.89-11.10)	China CDC National
30~	9.52 (7.96-11.08)	serosurvey 2006[17]
35~	7.77 (6.76-8.77)	
40~	8.58 (7.74-9.42)	

45~<br>50~<br>55~59	8.29 (6.50-10.08)<br>8.71 (6.65-10.77)<br>9.23 (6.94-11.52)	
**HBsAb** (%)<br>20~<br>25~<br>30~<br>35~<br>40~<br>45~<br>50~<br>55~59	 46.04 (40.90-51.19)<br>45.19 (42.51-47.88)<br>46.68 (44.03-49.32)<br>36.13 (43.50-48.76)<br>45.11 (42.66-47.56)<br>47.15 (43.16-51.15)<br>49.90 (45.84-53.97)<br>50.09 (47.61-54.27)	
**Data for calibration-**		
**year**	**Reported CHB incidence (numbers)**	
2006<br>2007<br>2008<br>2009<br>2010<br>2011<br>2012<br>2013<br>2014<br>2015<br>2016<br>2017<br>2018	1109130<br>1169946<br>1169569<br>1179607<br>1060582<br>1093335<br>1087086<br>962974<br>935702<br>934215<br>942268<br>1001952<br>999985	National Population and Health Science Data Sharing Platform of the Chinese Center for Disease Control and Prevention[18]

2.2 Initial values

Initial values for the population of the model compartments in 2006 are shown in Table S4, calculated based on total population size and the prevalence in 2006 mentioned above. The proportion for each health state was calculated according to a sero-survey among 1,400 CHB patients[19]. The initial values of compartments under diagnosis and treatment were assumed to be zero, which had little impact on the results.

Table S4 Initial population in compartments

State	18~	40~	50~	60~	70~	80~

Total population within age group	250988000	250632000	188589000	145921000	81431000	47337000
Susceptible	110069100	112715421	85856443	59469298	33186754	19291933
Acute HBV infection	0	0	0	0	0	0
Immunized	114507892	116291975	86796908	73432849	40979093	23821731
Immune Tolerant	1537036	1244695	623697	405545	160477	31893
Immune Active	10286316	8329883	4173969	2298090	1073962	287039
Immune Control	5333086	4318746	3772164	3067397	1405889	1100316
Immune Reactivation	7999630	6478119	6422875	6518218	4217668	2567404
Compensated Cirrhosis	746940	745881	561241	434261	242339	140875
Decompensated Cirrhosis	448265	447629	336820	260615	145436	84544
Hepatocellular Carcinoma	59735	59650	44884	34729	19381	11266

3 Intervention scenarios

The scenarios were set based on the community management strategies outlined in the 2020 Guidelines, involving vaccination, screening and diagnoses, treatment and monitoring follow-ups. The management process is as follows. Community mass screening was assumed to be conducted in a fixed proportion of the adult population by detecting the five hepatitis B markers (i.e., HBsAg, HbsAb, HbeAg, HbeAb and HbcAb or five-test). HBV seronegative individuals with antibodies are immune, and varying proportion of those without antibodies will receive 0-, 1-, and 6-month scheduled vaccinations under the vaccination strategies. Seropositive patients will receive further tests, such as the HBV DNA test and liver ultrasonography, to confirm HBV infection status. Treatment-eligible patients will take antiviral drugs, and GPs will monitor to ensure patient compliance [20], with coverage of treatment varying according to the scenario. And the reasons for choosing the four intervention scenarios are:

a) Universal vaccination. This scenario was designed from the perspective of protecting the susceptible population with consideration of simplicity of implementation (i.e., run an HBV vaccination campaign).
b) Screening and vaccination. This scenario was designed also from the perspective

of protecting the susceptible population while considering minimizing the intervention costs since the expense of screening is lower than that of vaccines. And this strategy might have some marginal health impact, that is, to raise disease awareness among infected individuals, which could enhance their diagnoses and treatment without promoting treatment through administrations.

c) Screening and treatment. This scenario was designed from the perspective of treating infected individuals and to investigate the potential impact of expanded screening and treatment which are assumed to have direct effect on HBV-related mortality.

d) Comprehensive interventions. This scenario was designed from the perspective of both protecting the susceptible and treating infected. The comprehensive strategy of screening, vaccination and treatment may have the largest health impact on Chinese adults.

4 Model outputs

4.1 Epidemiological outcomes

The total population in year *t* is:

$$N(t) = \sum_{\forall i} \sum_{\forall a} X_i(a,t) \tag{27}$$

The CHB prevalence of the whole population in year *t* is:

$$P(t) = \sum_{i=4}^{24} \sum_{\forall a} X_i(a,t) \,/\, \sum_{\forall i} \sum_{\forall a} X_i(a,t) \tag{28}$$

The number of new CHB cases in year *t* is:

$$C(t) = \sum_{\forall a} \sigma X_1(a,t) \lambda(a,t) \tag{29}$$

The number of HBV-related deaths in year *t* is:

$$D(t) = \sum_{i=16}^{24} \sum_{\forall a} X_i(a,t) \, \mu_n^k \tag{30}$$

The mortality rate of CHB in year *t* is:

$$M(t) = \sum_{i=16}^{24} \sum_{\forall a} X_i(a,t)\, \mu_n^k / total\_popu(t) * 100000 \tag{31}$$

μ_n^k in equations (30) and (31):

if $i \in (16,18)$, $n = 1$; if $i \in (19,21)$, $n = 2$; if $i \in (22,24)$, $n = 3$;

and k refers to the three states of cascade of care (U, D, T).

The time of elimination for absolute mortality target:

$$T_{absolute} = min\{t : M(t) < 4/100000\} - 2022 \tag{32}$$

The time of elimination for relative mortality target:

$$T_{relative} = min\{t : D(t) < 0.35 * D(t = 2015)\} - 2022 \tag{33}$$

4.2 Economic outcomes

We measured economic outcomes as QALYs, which were in turn based on cost calculations for the different interventions. All the economic outcomes were presented in per capita. These are calculated in equations listed below.

QALYs are defined using an integral expression to incorporate the discount rate *r*:

$$Q_i = \int_0^{45} \int_{18}^{80} e^{-rt} \sum_{\forall i} q_i X(a,t) da dt \tag{34}$$

Where here q_i measures the quality-of-life adjustment for population compartment *i* ($q_i \in [0,1]$). We calculated QALYs over the 45-year period from 2006 to 2050, with an annual discount rate of 3%.

The cost of universal vaccination is calculated with discount as:

$$C_{\text{vac1}} = \int_0^{45} \int_{18}^{80} e^{-rt} \sum_{\forall i} c_{vac} X_i(a,t) da dt \tag{35}$$

The cost of vaccination after screening is also discounted:

$$C_{\text{vac2}} = \int_0^{45} \int_{18}^{80} e^{-rt} c_{vac} X_1(a,t) da dt \tag{36}$$

As is the cost of screening:

$$C_{\text{scr}} = \int_0^{45} \int_{18}^{80} e^{-rt} \sum_{\forall i} c_{scr} X_i(a,t) dadt \tag{37}$$

As is the cost of diagnoses:

$$C_{diag} = \int_0^{45} \int_{18}^{80} \sum_{i=4,7,10,13,16,19,22} c_{diag} \delta^{\text{i}} X_i(a,t) dadt \tag{38}$$

The cost for treatment is defined in terms of the size under the CHB cascade of care *T* (diagnosed and on treatment), and is discounted in the same way:

$$C_{\text{treat}} = \int_0^{45} \int_{18}^{80} e^{-rt} \sum_{i=6,9,12,15,18,21} c_{treat} X_i(a,t) dadt \tag{39}$$

Finally, health care cost is given by:

$$C_{HC} = \int_0^{45} \int_{18}^{80} e^{-rt} \sum_{\forall i} c_{HC}^i X_i(a,t) dadt \tag{40}$$

5 Model calibration

We calibrated the model by comparing projected incidence values against observed values shown in Table S3. We first generated a sample of 1000 sets of parameter combination within the fluctuating range (±25%) of model parameters from a *Beta(2,2)* distribution. The model was run with this complete parameter set to obtain one set of annual epidemiological estimates. Ultimately, 1000 models with 1000 estimations were generated.

The projected values were compared with the observed data using a deviance-based loss:

$$D_S = 2 \sum_{\forall t} (Y_t ln(Y_t / \mu_{t,s}) - Y_t + \mu_{t,s}) \tag{41}$$

Where D_S is the deviance of the *s*th sample, $\mu_{t,s}$is the estimated prevalence in sample year t, and Y_t is the prevalence in year *t*. The 100 models with the lowest

deviance were retained as the final model set. The weighted mean of the 100 model was the final estimation, and the range of the 100 models formed the uncertainty range.

6 Supplemental results

6.1 Epidemic projections

Figure S2 shows the impact of different intervention scenarios on CHB epidemic and mortality over time with uncertainty range.

Table S5-S8 shows the mortality rate per 5 years from 2015 to 2080 under four scenarios, as well as the comparison to the relative and absolute elimination goals. Under *Comprehensive interventions*, the number of HBV-related deaths in 2050 was reduced by 68.52% compared to the 2015 baseline, which signified the achievement of the relative mortality target. The absolute target, however, was unlikely to be achieved in 30 years.

Table S5 Elimination analyses under *Universal vaccination*

Year	Relative mortality target		Absolute mortality target	
	Number of deaths (Uncertainty range)	Compared to 2015 (%)	Mortality rate/100,000 (Uncertainty range)	Compared to 4/100,000
2015	565281 (477204-665256)	/	52.88 (44.68-62.19)	Not met
2020	583513 (500696-677353)	103.23	52.55 (45.14-60.94)	Not met
2025	558427 (482027-643340)	98.79	48.92 (42.28-56.29)	Not met
2030	509937 (444007-583006)	90.21	43.83 (38.21-50.04)	Not met
2035	447761 (393659-508119)	79.21	38.00 (33.45-43.06)	Not met
2040	381495 (336046-429943)	67.49	32.12 (28.33-36.15)	Not met
2045	317942 (279977-356046)	56.24	26.65 (23.50-29.80)	Not met
2050	260424 (228843-289883)	46.07	21.78 (19.16-24.21)	Not met
2055	199756 (175038-223916)	35.34	16.70 (14.65-18.70)	Not met
2060	158506 (139151-178070)	28.04	13.25 (11.64-14.87)	Not met
2065	124298 (109235-139782)	21.99	10.39 (9.14-11.67)	Not met
2070	96487 (84884-108491)	17.07	8.07 (7.10-9.07)	Not met
2075	74272 (65509-83409)	13.14	6.21 (5.48-6.97)	Not met
2080	56806 (50237-63954)	10.05	4.75 (4.21-5.35)	Not met

Table S6 Elimination analyses under *Screening and vaccination*

Year	Relative mortality target		Absolute mortality target	

	Number of deaths (Uncertainty range)	Compared to 2015 (%)	Mortality rate/100,000 (Uncertainty range)	Compared to 4/100,000
2015	565281 (477204-665256)	/	52.88 (44.68-62.19)	Not met
2020	583513 (500696-677353)	103.23	52.55 (45.14-60.94)	Not met
2025	558406 (482004-643316)	98.78	48.92 (42.28-56.29)	Not met
2030	508997 (443191-581889)	90.04	43.75 (38.14-49.94)	Not met
2035	444906 (391207-504751)	78.71	37.76 (33.24-42.77)	Not met
2040	376468 (331392-424078)	66.6	31.70 (27.94-35.65)	Not met
2045	311077 (273670-348129)	55.03	26.07 (22.97-29.14)	Not met
2050	252345 (221620-280669)	44.64	21.10 (18.56-23.44)	Not met
2055	191502 (167866-214360)	33.88	16.01 (14.05-17.90)	Not met
2060	150302 (131992-168552)	26.59	12.56 (11.04-14.07)	Not met
2065	116556 (102397-130792)	20.62	9.74 (8.57-10.92)	Not met
2070	89466 (78693-100474)	15.83	7.48 (6.59-8.40)	Not met
2075	68099 (60062-76634)	12.05	5.70 (5.03-6.41)	Not met
2080	51506 (45605-58137)	9.11	4.31 (3.82-4.86)	Not met

Table S7 Elimination analyses under *Screening and treatment*

Year	Relative mortality target		Absolute mortality target	
	Number of deaths (Uncertainty range)	Compared to 2015 (%)	Mortality rate/100,000 (Uncertainty range)	Compared to 4/100,000
2015	565281 (477204-665256)	/	52.88 (44.68-62.19)	Not met
2020	583513 (500696-677353)	103.23	52.55 (45.14-60.94)	Not met
2025	551764 (476287-636160)	97.61	48.34 (41.78-55.66)	Not met
2030	483291 (421277-552336)	85.5	41.54 (36.26-47.40)	Not met
2035	400497 (352452-453071)	70.85	33.99 (29.95-38.39)	Not met
2040	318024 (278718-356323)	56.26	26.78 (23.50-29.96)	Not met
2045	244982 (214186-272910)	43.34	20.53 (17.98-22.84)	Not met
2050	184888 (161470-206613)	32.71	15.46 (13.52-17.26)	Not met
2055	129081 (113632-146188)	22.83	10.79 (9.51-12.21)	Not met
2060	94595 (83256-107528)	16.73	7.91 (6.97-8.98)	Not met
2065	68850 (60606-78499)	12.18	5.76 (5.07-6.56)	Not met
2070	49857 (43911-57005)	8.82	4.17 (3.68-4.76)	Not met
2075	35954 (31698-41232)	6.36	3.01 (2.65-3.45)	Met
2080	25835 (22810-29728)	4.57	2.16 (1.91-2.49)	Met

Table S8 Elimination analyses under *Comprehensive interventions*

Year	Relative mortality target		Absolute mortality target	
	Number of deaths (Uncertainty range)	Compared to 2015 (%)	Mortality rate/100,000 (Uncertainty range)	Compared to 4/100,000
2015	565,281 (477,204-665,256)	/	52.88 (44.68-62.19)	Not met

2020	583513 (500696-677353)	103.23	52.55 (45.14-60.94)	Not met
2025	551,764 (476,287-636,160)	97.61	48.34 (41.78-55.66)	Not met
2030	482,346 (420,488-551,207)	85.33	41.45 (36.19-47.31)	Not met
2035	396,128 (348,133-447,993)	70.08	33.62 (29.58-37.96)	Not met
2040	310,400 (271,280-347,652)	54.91	26.13 (22.87-29.23)	Not met
2045	235,934 (205,524-263,709)	41.74	19.77 (17.25-22.07)	Not met
2050	175,989 (153,119-197,323)	31.13	14.72 (12.82-16.48)	Not met
2055	122753 (107949-139086)	21.72	10.26 (9.03-11.61)	Not met
2060	89358 (78645-101530)	15.81	7.47 (6.58-8.48)	Not met
2065	64681 (56997-73641)	11.44	5.41 (4.77-6.15)	Not met
2070	46614 (41114-53166)	8.25	3.90 (3.44-4.44)	Met
2075	33465 (29558-38243)	5.92	2.80 (2.47-3.20)	Met
2080	23939 (21184-27420)	4.23	2.00 (1.77-2.29)	Met

6.2 Cost-effectiveness analysis

Fig. S3-S5 supplemented the quantitative details of the one-way sensitivity results of average CER under scenarios *Universal vaccination* and *Screening and vaccination*. The ten parameters most influential on average CER was listed beside the tornado in descending order. The three scenarios would remain cost-effective over the wide variety of all parameters.

7 CHEERS checklist

The Consolidated Health Economic Evaluation Reporting Standards (CHEERS) Checklist contains 28 items evaluating an economic analysis[21] (Also available on the ISPOR webpage: https://www.ispor.org/heor-resources/good-practices/cheers). And our analysis was described according to the checklist below in Table S9.

Table S9 CHEERS checklist

Section	No.	Recommendation	Location where item is reported
**Title**			
Title	1	Identify the study as an economic evaluation and specify the interventions being compared.	Title page
**Abstract**			
Abstract	2	Provide a structured summary that highlights context, key methods, results, and alternative analyses.	Abstract
**Introduction**			

Background and objectives	3	Give the context for the study, the study question, and its practical relevance for decision making in policy or practice.	Introduction, 1st to 4th paragraph
**Methods**			
Health economic analysis plan	4	Indicate whether a health economic analysis plan was developed and where available.	Methods, Cost-effectiveness analysis
Study population	5	Describe characteristics of the study population (such as age range, demographics, socioeconomic, or clinical characteristics).	Methods, Model overview, 1st paragraph
Setting and location	6	Provide relevant contextual information that may influence findings.	Methods, Model overview, 2nd paragraph
Comparators	7	Describe the interventions or strategies being compared and why chosen.	Methods, Intervention scenarios
Perspective	8	State the perspective(s) adopted by the study and why chosen.	Methods, Cost-effectiveness analysis
Time horizon	9	State the time horizon for the study and why appropriate.	Methods, Model overview, 1st paragraph
Discount rate	10	Report the discount rate(s) and reason chosen.	Methods, Cost-effectiveness analysis
Selection of outcomes	11	Describe what outcomes were used as the measure(s) of benefit(s) and harm(s).	Supplementary Section 4
Measurement of outcomes	12	Describe how outcomes used to capture benefit(s) and harm(s) were measured.	Supplementary Section 4
Valuation of outcomes	13	Describe the population and methods used to measure and value outcomes.	Methods, Model overview
Measurement and valuation of resources and costs	14	Describe how costs were valued.	Supplementary Section 2
Currency, price date, and conversion	15	Report the dates of the estimated resource quantities and unit costs, plus the currency and year of conversion.	Not Reported
Rationale and description of model	16	If modelling is used, describe in detail and why used. Report if the model is publicly available and where it can be accessed.	Introduction, 4th paragraph; Methods, Model overview
Analytics and assumptions	17	Describe any methods for analyzing or statistically transforming data, any extrapolation methods, and approaches for validating any model used.	Methods, Model calibration
Characterizing heterogeneity	18	Describe any methods used for estimating how the results of the study vary for subgroups.	Methods, Intervention scenarios
Characterizing distributional effects	19	Describe how impacts are distributed across different individuals or adjustments made to reflect priority populations.	Not applicable

Characterizing uncertainty	20	Describe methods to characterize any sources of uncertainty in the analysis.	Methods, Sensitivity analysis
Approach to engagement with patients and others affected by the study	21	Describe any approaches to engage patients or service recipients, the general public, communities, or stakeholders (such as clinicians or payers) in the design of the study.	Not applicable
**Results**			
Study parameters	22	Report all analytic inputs (such as values, ranges, references) including uncertainty or distributional assumptions.	Supplementary Table S2; Methods, Model calibration
Summary of main results	23	Report the mean values for the main categories of costs and outcomes of interest and summarize them in the most appropriate overall measure.	Results, Cost-effectiveness analyses
Effect of uncertainty	24	Describe how uncertainty about analytic judgments, inputs, or projections affect findings. Report the effect of choice of discount rate and time horizon, if applicable.	Results, Sensitivity analyses
Effect of engagement with patients and others affected by the study	25	Report on any difference patient/service recipient, general public, community, or stakeholder involvement made to the approach or findings of the study	Not applicable.
**Discussion**			
Study findings, limitations, generalisability, and current knowledge	26	Report key findings, limitations, ethical or equity considerations not captured, and how these could affect patients, policy, or practice.	Discussion
**Other relevant information**			
Source of funding	27	Describe how the study was funded and any role of the funder in the identification, design, conduct, and reporting of the analysis	Declarations, Funding
Conflicts of interest	28	Report authors conflicts of interest according to journal or International Committee of Medical Journal Editors requirements.	Declarations, Conflict of Interests

8 References

1. Edmunds, W.J., et al., *The influence of age on the development of the hepatitis B carrier state.* Proc Biol Sci, 1993. **253**(1337): p. 197-201.
2. McCulloch, K., et al., *Modeling Progress Toward Elimination of Hepatitis B in Australia.* Hepatology, 2020. **71**(4): p. 1170-1181.

3. de Villiers, M.J., et al., *Modelling hepatitis B virus infection and impact of timely birth dose vaccine: A comparison of two simulation models.* PLoS One, 2020. **15**(8): p. e0237525.
4. Kim, H.L., et al., *Cost-effectiveness of antiviral treatment in adult patients with immune-tolerant phase chronic hepatitis B.* Gut, 2020.
5. Yang, P.C., et al., *[Development of Markov models for economics evaluation of strategies on hepatitis B vaccination and population-based antiviral treatment in China].* Zhonghua Liu Xing Bing Xue Za Zhi, 2017. **38**(7): p. 845-851.
6. Nayagam, S., et al., *Requirements for global elimination of hepatitis B: a modelling study.* The Lancet Infectious Diseases, 2016. **16**(12): p. 1399-1408.
7. Toy, M., D.W. Hutton, and S. So, *Population Health And Economic Impacts Of Reaching Chronic Hepatitis B Diagnosis And Treatment Targets In The US.* Health Aff (Millwood), 2018. **37**(7): p. 1033-1040.
8. Lin, X., et al., *Chronic hepatitis B virus infection in the Asia-Pacific region and Africa: review of disease progression.* J Gastroenterol Hepatol, 2005. **20**(6): p. 833-43.
9. Fattovich, G., F. Bortolotti, and F. Donato, *Natural history of chronic hepatitis B: special emphasis on disease progression and prognostic factors.* J Hepatol, 2008. **48**(2): p. 335-52.
10. Chen, J.D., et al., *Carriers of inactive hepatitis B virus are still at risk for hepatocellular carcinoma and liver-related death.* Gastroenterology, 2010. **138**(5): p. 1747-54.
11. *United Nations, Department of Economic and Social Affairs, Population Division (2022). World Population Prospects 2022, Online Edition.*
12. Wu, Z., et al., *Suitable hepatitis B vaccine for adult immunization in China: a systematic review and meta-analysis.* Hum Vaccin Immunother, 2019. **15**(1): p. 220-227.
13. Levy, A.R., et al., *The impact of chronic hepatitis B on quality of life: a multinational study of utilities from infected and uninfected persons.* Value Health, 2008. **11**(3): p. 527-38.
14. Rossi, C., et al., *Hepatitis B screening and vaccination strategies for newly arrived adult Canadian immigrants and refugees: a cost-effectiveness analysis.* PLoS One, 2013. **8**(10): p. e78548.
15. Hu, M. and W. Chen, *Assessment of total economic burden of chronic hepatitis B (CHB)-related diseases in Beijing and Guangzhou, China.* Value Health, 2009. **12 Suppl 3**: p. S89-92.
16. Li, J., et al., *A mathematical model of biomedical interventions for HIV prevention among men who have sex with men in China.* BMC Infect Dis, 2018. **18**(1): p. 600.
17. Disease Control Bureau, Ministry of Health, and Chinese Disease Prevention and Control Center, *The National Report of Seroepidemiologic investigation of hepatitis B in China*. 2011, Beijing: People's Medical Publishing House.

18. Chinese Center for Disease Control and Prevention. *National Population and Health Science Data Sharing Platform-Infectious Diseases-Hepatitis*. 2022 2022-04-01]; Available from: https://www.phsciencedata.cn/Share/en/index.jsp.
19. Fung, J., et al., *Profiles of HBV DNA in a large population of Chinese patients with chronic hepatitis B: implications for antiviral therapy.* J Hepatol, 2011. **54**(2): p. 195-200.
20. Chinese Medical Association, et al., *Guideline for primary care of chironic hepatiis B (2020).* Chinese Journal of General Practitioners, 2020. **20**: p. 137-149.
21. Husereau, D., et al., *Consolidated Health Economic Evaluation Reporting Standards (CHEERS) 2022 Explanation and Elaboration: A Report of the ISPOR CHEERS II Good Practices Task Force.* Value Health, 2022. **25**(1): p. 10-31.

Figures

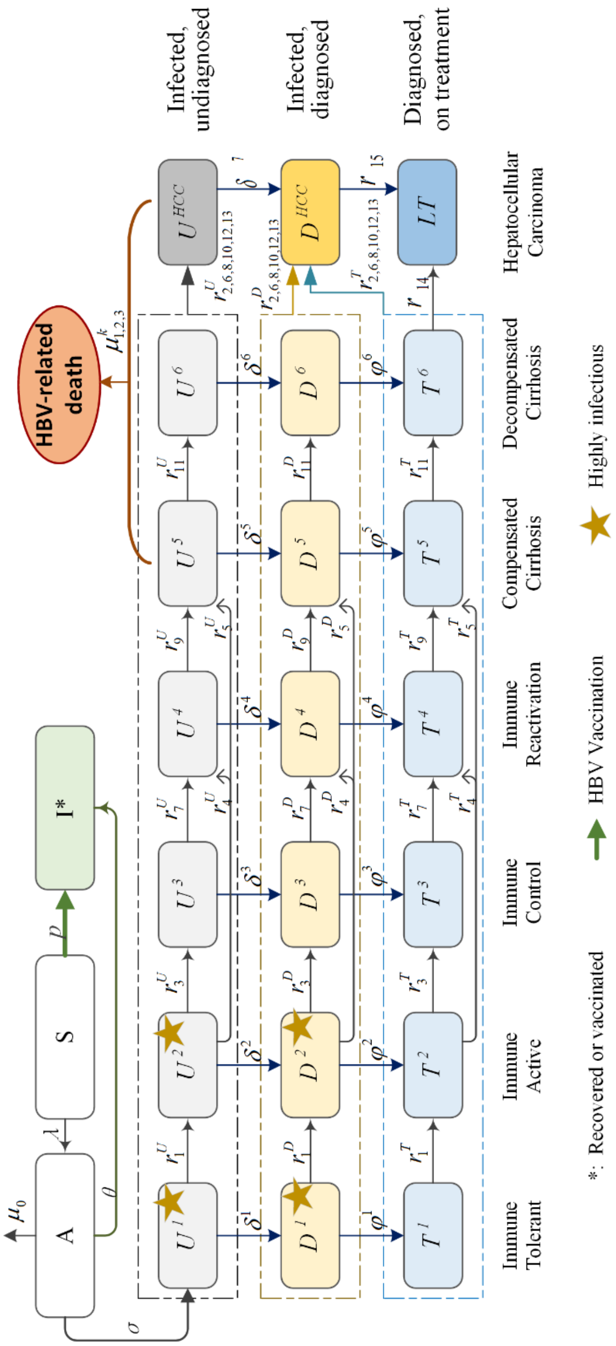

Fig. S1 HBV compartmental model diagram with parameters. The compartments are denoted by the variables S (susceptible), A (acute HBV infection), I (immunized), U (infected but undiagnosed), D (diagnosed), T (diagnosed and on treatment). Superscripts of the U, D, and T compartments represent CHB phases. Solid black arrows represent disease progression while colored shades denote the community-based cascade of care. Detailed model structure and transition rates are given in Table S2. Abbreviation: HBV, hepatitis B virus.

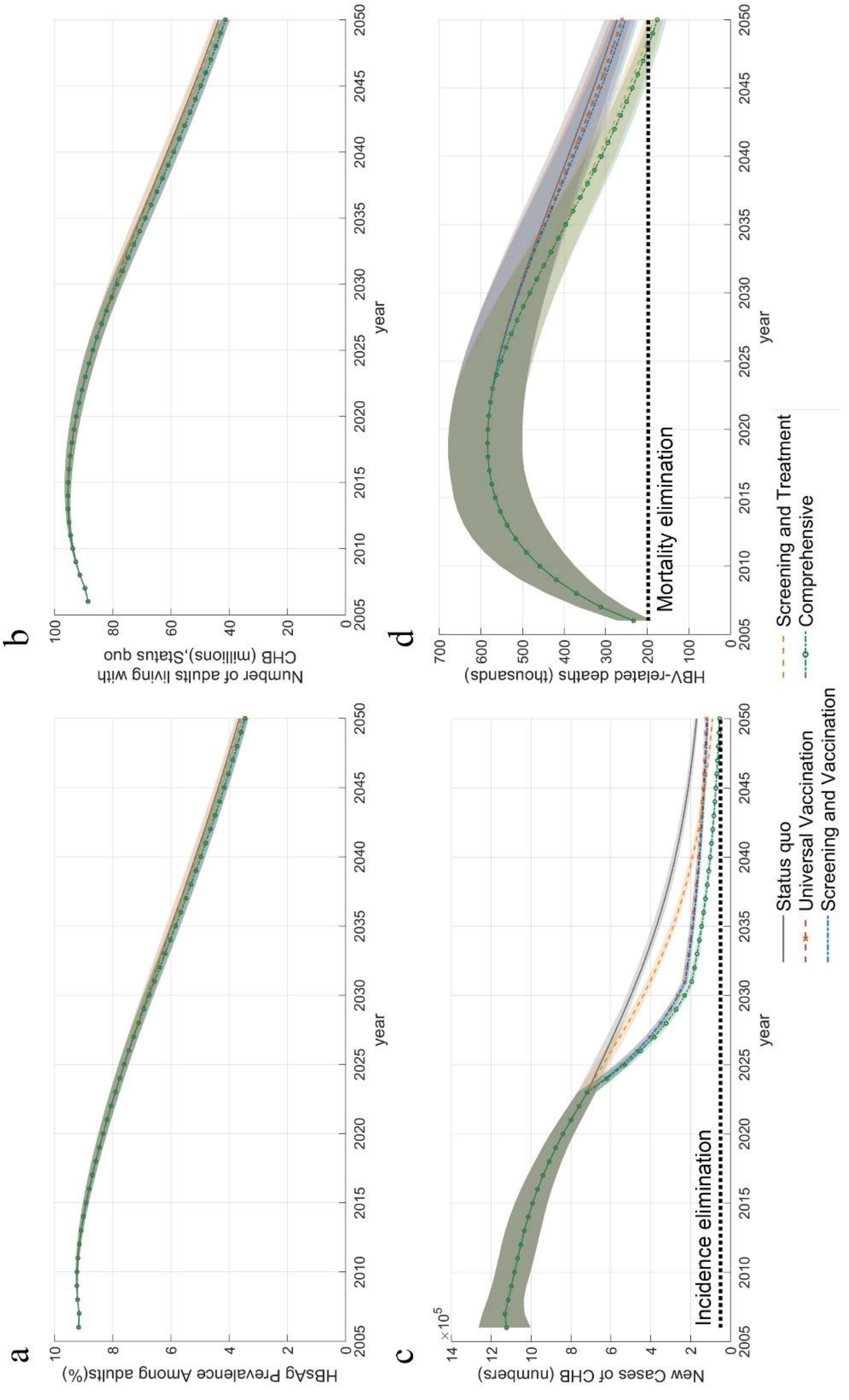

Fig. S2 Impact (with uncertainty range) of four community-based intervention scenarios on(a) HBsAg prevalence, (b) Number of people living with HBV, (c) CHB incidence, and (d) HBV-related deaths among adults. Abbreviation: HBV hepatitis B virus, CHB chronic hepatitis B.

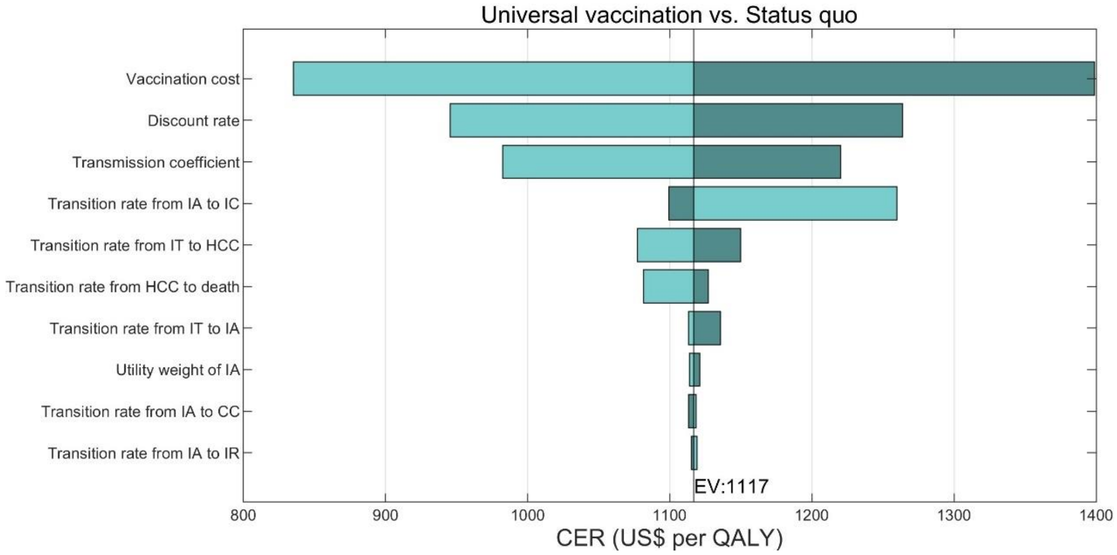

Fig. S3 One-way sensitivity analysis of average CER (*Universal vaccination* vs. the Status quo). Abbreviation: QALY quality-adjusted life years, IT immune tolerant, IA immune active, IC immune control, IR immune reactivation, CC compensated Cirrhosis, DC decompensated Cirrhosis, HCC hepatocellular carcinoma.

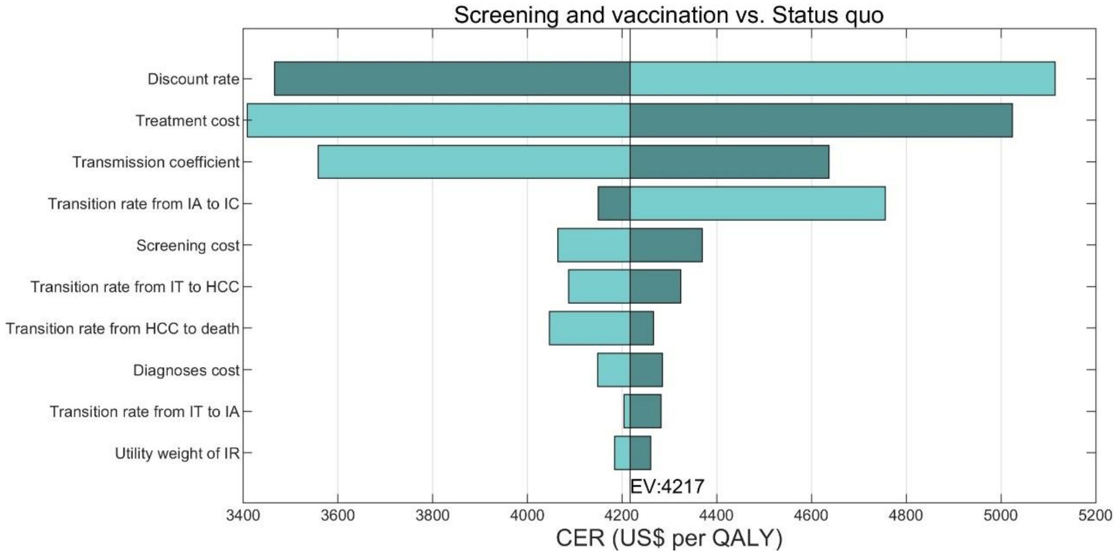

Fig. S4 One-way sensitivity analysis of average CER (*Screening and vaccination* vs. the Status quo). Abbreviation: QALY quality-adjusted life years, IT immune tolerant, IA immune active, IC immune control, HCC hepatocellular carcinoma.

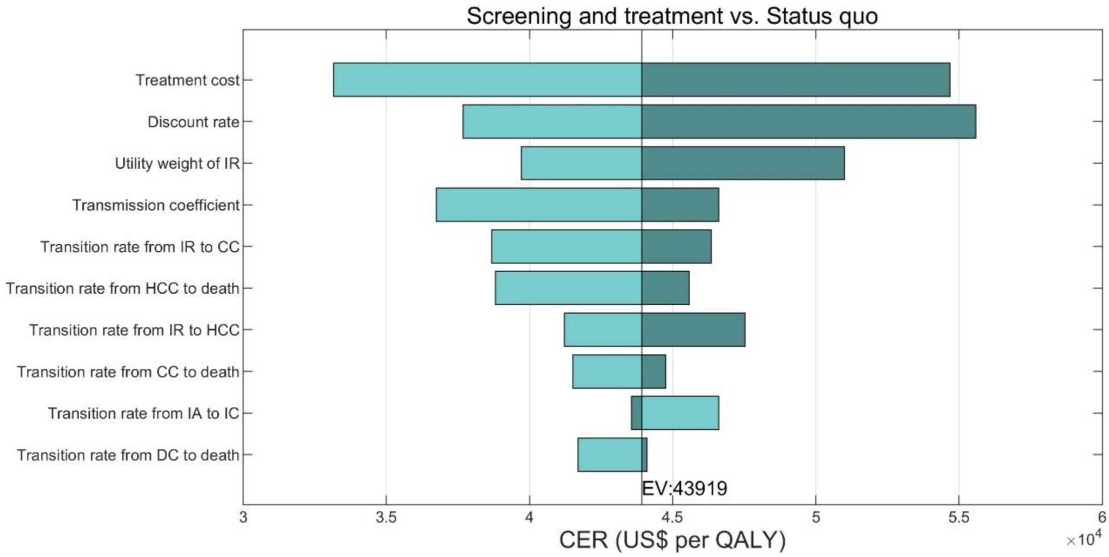

Fig. S5 One-way sensitivity analysis of average CER (*Screening and treatment* vs. the Status quo). Abbreviation: QALY quality-adjusted life years, IA immune active, IC immune control, IR immune reactivation, CC compensated Cirrhosis, DC decompensated Cirrhosis, HCC hepatocellular carcinoma.
